# Dr. Chi-Ming Chu: Respected founder of molecular virology and pioneer of biologicals in China

**DOI:** 10.1007/s13238-017-0445-z

**Published:** 2017-07-28

**Authors:** Weizheng Yan, Baoying Huang, Li Ruan, Wenjie Tan

**Affiliations:** Biotech Center for Viral Diseases Emergency, Key Laboratory of Medical Virology, Ministry of Health, National Institute for Viral Disease Control and Prevention, China CDC, Beijing, 102206 China

***“I have the greatest respect for Dr. Chu, who upheld great standards of science during very difficult times in China. His contributions to the genetic characterisation of influenza viruses were extraordinary.”***

—Professor Peter Palese, a member of the National Academy of Sciences and expert in the field of RNA viruses, wrote this when he received the obituary notice of Dr. Chu in 1998.

***“He was not only a great scientist and teacher, but also a remarkable human being who contributed significantly to openness in science and international relations.”***

—Professors Graeme Laver and Robert Webster, famous virologists at the Department of Virology and Molecular Biology (St. Jude’s Children’s Research Hospital, USA), wrote an obituary for Dr. Chu in 1999 (Laver and Webster, 1999).

Dr. Chi-Ming Chu (Ji-Ming Chu, 朱既明) was an internationally renowned virologist, one of the most important founders of molecular virology and a pioneer of biologicals in China (Fig. 1). He was a leader in virology research in China, from classical virology to the era of molecular virology. His seminal observations and research on influenza viruses laid the foundation for worldwide research on influenza virus structure, surveillance and subunit vaccine development. He was a pioneer in the field of biologicals in China due to his extraordinary contributions to research on penicillin, attenuated viral vaccines, recombinant hepatitis B virus (HBV) vaccines and vaccinia viral vectors.

**Figure 1:**
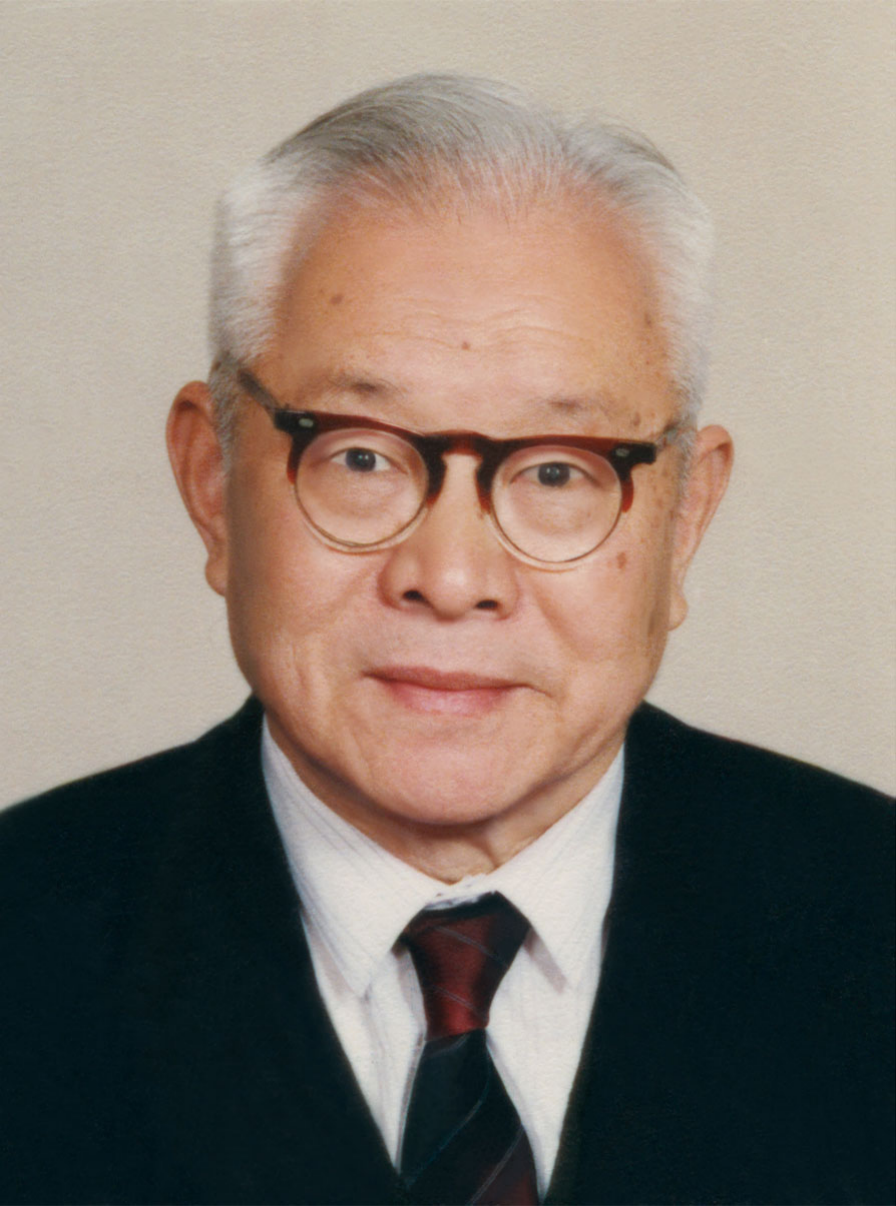
Dr. Chi-Ming Chu (1917.9.12–1998.1.6)

Dr. Chu was born on September 12, 1917, in Yixing County, Jiangsu Province. He graduated from Shanghai Medical College in 1939 and was awarded a Ph.D. by Cambridge University (UK) in 1948. He worked for the World Influenza Centre at the National Institute for Medical Research at Mill Hill (London) from 1948 to 1950. After returning to China in 1950, he worked successively at the National Vaccine and Serum Institute (Beijing), National Institute of Biologicals in Changchun and the Chinese Academy of Preventive Medicine (CAPM) (Ruan and Lu, 1998; Zhang, 1998). At the time of his death on January 6, 1998, Dr. Chu was a member of the Chinese Academy of Science, an Academician of the British Royal Institute for Internal Medicine, an Honorary Member of the American Society for Microbiology and Honorary Director of the Institute of Virology (CAPM). He was elected as a member of the National People’s Congress from 1964 to 1974 and was a member of the fifth, sixth, and seventh Chinese People’s Political Consultative Conferences from 1978 to 1992 (Ruan, 2013).

## Dr. Chu made an extraordinary contribution to the development of Chinese biologicals

Penicillin was a magical drug saved thousands and millions people’s life during the Second World War. Dr. Chu contributed significantly to the development of China’s first antibiotic of penicillin (Ruan, 2015). From 1940 to 1945, Dr. Chu worked at the National Epidemic Prevention Bureau (NEPB) in Kunming under Professor Tang Feifan’s direction, cooperated together with Dr. Fan Qingsheng, a prominent agricultural microbiologist at the time, Dr. Chu and his colleagues compared 30 local strains and some foreign strains, and finally selected the local No. 22 strain for the extraction and purification of penicillin (Chu et al., 1945; Xu, 2013). A seed strain of lyophilized penicillin for injection was successfully developed and the preliminary clinical observation was performed by Dr. Chu’s group. This work contributed significantly as the beginning of antibiotics research and application in China.

Beginning in the 1950s, Dr. Chu conducted research on live attenuated vaccines. Specifically, he studied forest encephalitis, measles, and influenza live vaccines. During his research on the live influenza vaccine, he found that the rate of attenuation of influenza viruses differs yearly, and he developed two methods for producing live vaccines: chick embryo passage and genetic recombination. In 1959, a measles pandemic occurred in China, and Dr. Chu and his colleagues in Changchun began to study live attenuated measles vaccines. After 6 years of hard work, he, together with his colleagues, selected the *Chang 47* measles strain; the vaccine produced using this strain had mild adverse reactions, a high seroconversion rate and induced high-titre antibody responses (Chu, 1964). The vaccine produced from this strain in 1966 made an important contribution to the control of measles in China.

From 1951 to 1963, Dr. Chu was appointed Chief of the Second Research Laboratory, Chief of the Quality Control Department of the National Vaccine and Serum Institute, and Chief of the Biological Product Training Class conducted by the Ministry of Health of China. Subsequently, he became Deputy Director of the National Institute of Biological Products in Changchun (Zhang, 1998), and Deputy Director and General Director of the Institute of Virology (CAPM). He wrote the first biological product regulation draft in Chinese and trained groups of young scientists (Ruan, 2013).

Dr. Chu promoted research on vaccinia viral vectors in China. At the start of 1984, he directed the genetic engineering of a polyvalent vaccine vectored by the Tiantan vaccinia virus strain, with the aim of developing a vaccine effective against several diseases. He designed a polyvalent vaccinia vaccine expressing antigens from HBV, Epstein-Barr virus, and herpes simplex virus (Tsao et al., 1988). This repertoire was later expanded to include antigens of hepatitis A virus, measles virus, and respiratory syncytial virus. This work was supported by the National High-Technology Research and Development Program (863 program) in 1986. Under his direction, the Tiantan vaccinia virus strain was used for the genetic engineering of antigen expression, resulting in clinical trials of several recombinant vaccine candidates. In addition, he initiated and guided the R&D program of a non-replicating vaccinia virus vector (Tiantan strain) with the intellectual property rights for Chinese, which was subsequently used extensively in vaccine research in China.

Hepatitis B has had a major impact on the health of the Chinese. In the 1980s, it became clear that a new HBV vaccine was needed. Dr. Chu contributed significantly to the first genetically engineered recombinant HBV vaccine. He undertook research into a recombinant hepatitis B vaccine expressed in Chinese hamster ovary (CHO) cells, assuming directorship of the project. After more than 10 years of hard work, he and his colleagues finally succeeded in developing a genetically engineered hepatitis B vaccine expressed in CHO cells, which was the first genetically engineered HBV vaccine produced in CHO cells (Ruan et al., 1983). The vaccine has been produced since 1991 and has played an important role in the control of HBV transmission in China.

## Dr. Chu was one of the greatest influenza research scientists in the world

Dr. Chu was engaged in influenza virus research from 1945 to 1984. Among his contributions, Dr. Chu discovered the filamentous form of influenza virus and was the first to report its variability in *Lancet* in 1949 (Chu et al., 1949); his findings were later confirmed by other scientists. During the same period, he found that beta- and gamma-inhibitors could inhibit the agglutination of red blood cells by influenza virus (Chu et al., 1947; Chu, 1948); the beta-inhibitor is also termed Chu’s inhibitor. Subsequently, he discovered that influenza virus could be divided into two subunits by treatment with ether or detergents: haemagglutinin and soluble antigens. This led to the determination of the structure of the virus in the 1950s and subsequently to the development of a subunit vaccine.

Antigenic shifts in influenza viruses were discovered by Dr. Chu. He also discovered the origin of the 1957 and 1977 influenza pandemics (Laver and Webster, 1999). In 1949, Dr. Chu demonstrated that a qualitative change in influenza virus antigens (drift) subsequently spreads to other locations (Chu, 1951). In February of 1957, the H2N2 Asian pandemic strain appeared in Kweiyang, Kweichow Province, Southern China, and spread to all of China by the end of March, replacing the previously prevalent H1N1 strains. Dr. Chu showed that this pandemic influenza virus was a new subtype resulting from a qualitative change in the haemagglutinin antigen (i.e., antigenic shift). This was the first pandemic caused by a new subtype that was proven virologically. Following this, he showed the re-emergence of the 1950 H1N1 strain in Anshan in February of 1977, 9 months before it reached the rest of the world and became known as “Russian influenza” (Laver, et al, 1984). The concept of quantitative changes in influenza virus antigens subsequently became a guiding principle for flu monitoring and vaccine seed selection.

Influenza temperature-sensitive strains in nature were firstly discovered by Dr. Chu, who was the first scientist to show that influenza virus undergoes not only antigenic variation but also virulence variation. In 1977, he found that the newly isolated influenza virus strains could be divided into normal and temperature-sensitive types (Chu et al., 1982). Temperature-sensitive strains can reproduce only at 33°C and are not pathogenic to humans. Genetic research showed that temperature sensitivity could be transferred to a normal strain by genetic recombination (Chu et al., 1992). This suggested the possibility of isolating attenuated strains in the field for use in live vaccines.

## Dr. Chu was always courteous as well as a master of knowledge; a worthy mentor

**Figure 2:**
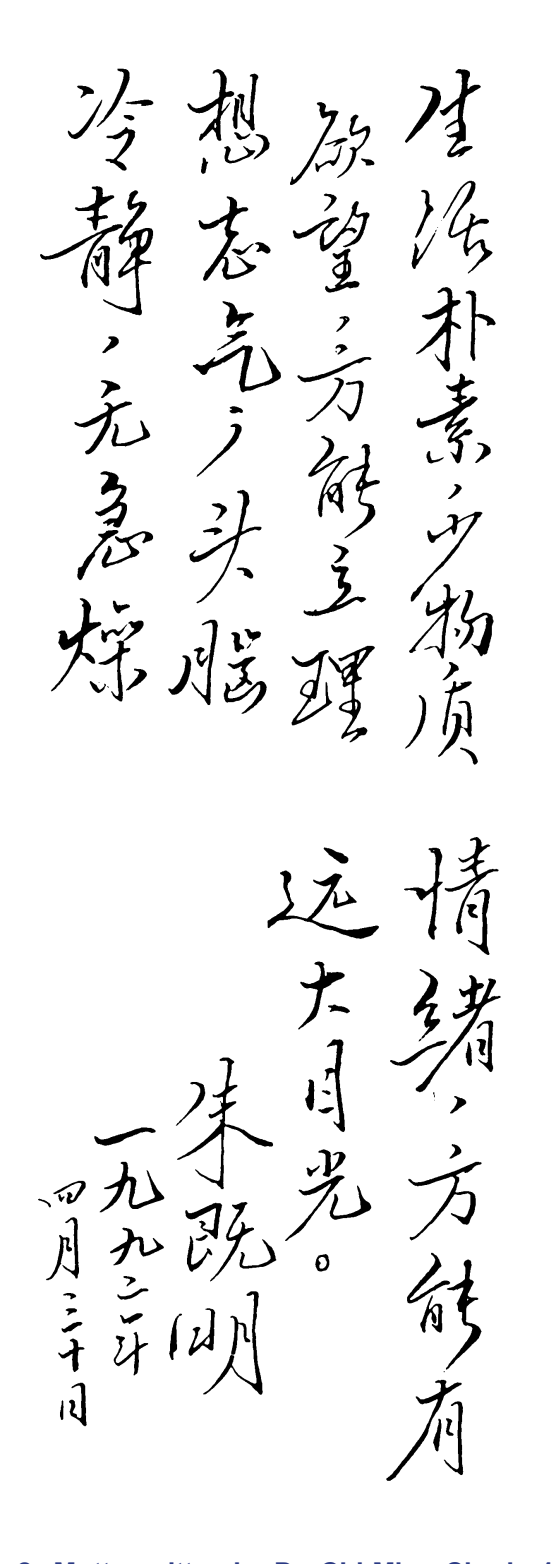
Motto written by Dr. Chi-Ming Chu in 1992

This is the personal motto of Dr. Chu: “Only by simplifying your everyday life and suppressing your desire for material things can you reach your goals. Only a calm mind and absence of anxiety will grant you broad vision” (Fig. 2). He stressed the importance of integrity to his students and to himself. He was upright and lived simply, and was not motivated by fame or wealth. He was rigorous in both scientific research and in his life. He was not only a great scientist but also a remarkable human being. Dr. Chu will be remembered for his great contributions and nobility of character, and we who knew him will miss him forever.

Dr. Chu was always keen to keep up with international developments in science and technology. He encouraged his colleagues and students to adopt a strategic perspective and think innovatively; he emphasised putting ideas into practice, and he encouraged collaboration and long-term efforts to overcome major difficulties. He repeatedly proposed creative solutions to key issues in the prevention and control of viral diseases in China. In the early 1970s, he reviewed global developments in virology research and realised that the era of molecular virology was approaching. After visiting several American laboratories engaged in viral molecular genetics and genetic engineering research in 1978, he proposed and actively supported genetic engineering research in China and established the laboratory of viral genetics (The predecessor of Biotech Center for Viral Diseases Emergency, China CDC now) at the Institute of Virology (CAPM). In 1981, he published a paper titled *Virus and Recombinant DNA* and proposed two research directions for the laboratory: gene expression in CHO cells and the virus as a vector for genetic engineering. This marked a change in his research focus from classic to molecular virology.

**Figure 3:**
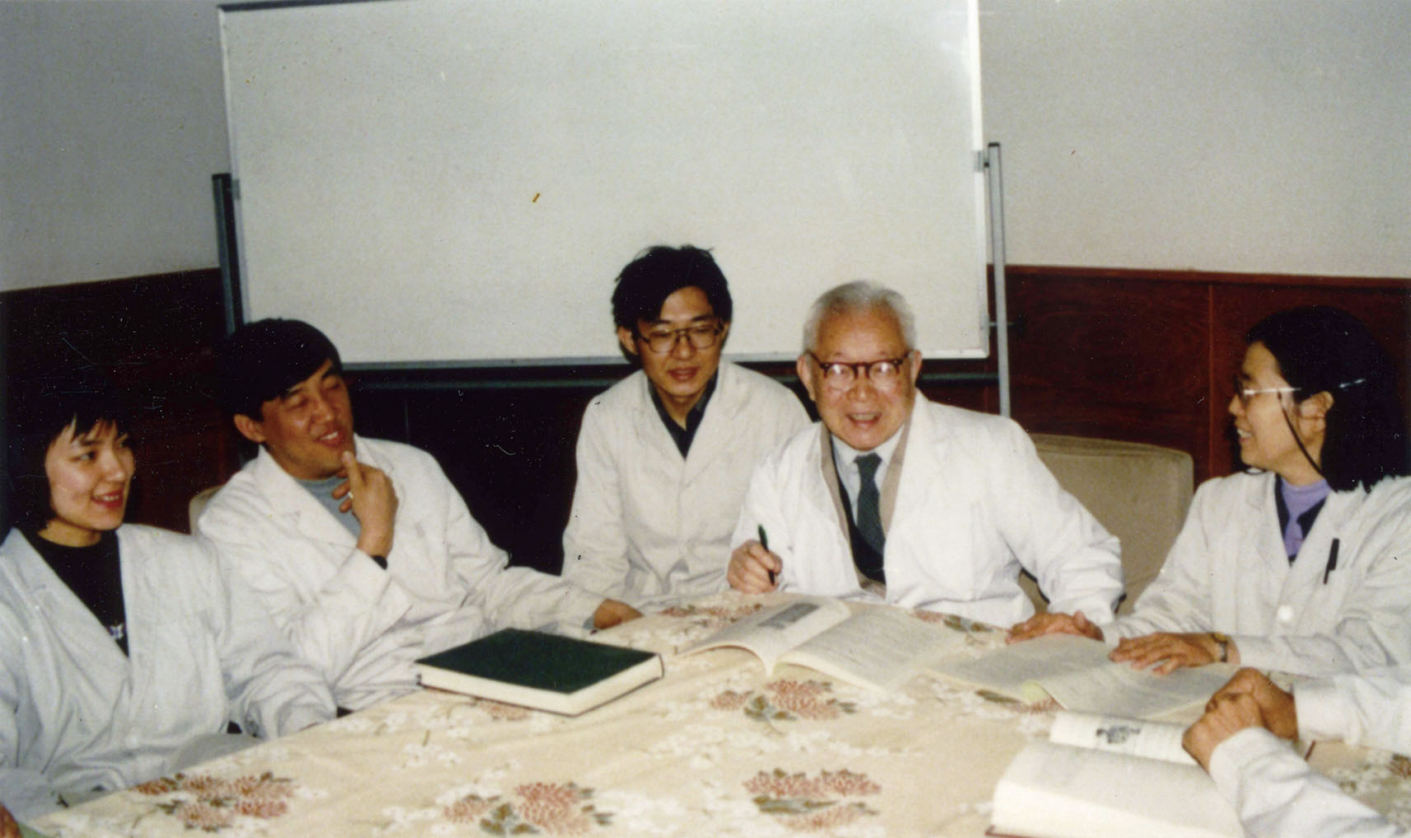
Dr. Chi-Ming Chu (second on the right) discussing scientific topics with colleagues at a laboratory meeting in the Institute of Virology, CAPM (now renamed as Institute for Vial Disease Control and Prevention, China CDC) in 1994

As the founder of the Chinese journal *Virology* (*Bing du xue bao*), Dr. Chu made specific comments on each article accepted by this journal, such as on academic issues, English usage and illustrations. He made the final decision to accept or reject a manuscript after peer review based on academic quality (Jin, 2009). He personally instructed colleagues and students, often attending academic activities in laboratories and institutes, discussing issues in a rigorous manner and encouraging innovation (Fig. 3). He took the initiative to encourage the younger generation, for entirely selfless reasons.

The internationally famous influenza virologists Graeme Laver and Robert Webster described their first meeting with Dr. Chu (Laver and Webster, 1999): “We first met Professor Chu in 1972. He had come to the Beijing railway station at 4:00 AM to meet our visiting Australian medical delegation. We were most impressed that such a prestigious scientist would find the time to welcome us to China, especially at such an early hour and so soon after the ‘Cultural Revolution’. This courtesy was typical of Dr. Chu.” Dr. Chu also lived like a British gentleman; he was unfailingly neatly dressed with meticulously looked-after hair. He treated everyone with modesty and kindness.

Based his distinguished contributions to virology and biologicals, Dr. Chu was honoured with the National Science Conference Award, the First-Class Award for Scientific Advancement by the State, the Third-Class Prize in Natural Science of the State, and the First-Class Award for Scientific Advancement by the Ministry of Health, and the Ho Leung Ho Lee Scientific Advancement in Medical Science Award. he was invited to be an adviser to the international journal *Archive of Virology* from 1982 to 1988, served as chief editor of the *Chinese Journal of Virology*, as deputy editor of the *Journal of Microbiology and Immunology*, the *Chinese Journal of Preventive Medicine* and the *Journal of Tropical Medicine*, and he was on the editorial boards of the *National Medical Journal of China* (English Edition), *Acta Microbiologica Sinica* and the *Chinese Journal of Epidemiology*.

Dr. Chu was a truly international Chinese virologist who made significant contributions to solving key problems in viral disease control in China. Dr. Chu has died, but his spirit and style are eternal. We, his former colleagues and students, penned this memorial on the occasion of the centennial of Dr. Chu’s birthday. We try always to emulate Dr. Chu’s spirit, courage, enthusiasm, meticulousness and dedication, and to be worthy recipients of his legacy.
